# Co-designing strategies to implement long-acting injectable PrEP for sexual minority men in Chicago: a study protocol for an innovation tournament and implementation mapping

**DOI:** 10.1186/s43058-024-00574-z

**Published:** 2024-03-25

**Authors:** Amelia E. Van Pelt, Elizabeth Casline, Gregory Phillips, Jorge Cestou, Brian Mustanski, Grace Cook, Rinad S. Beidas

**Affiliations:** 1 Department of Medical Social Sciences, Northwestern University Feinberg School of Medicine, 625 N Michigan Ave Suite 2100, Chicago, IL 60611 USA; 2 Center for Dissemination and Implementation Science, Northwestern University Feinberg School of Medicine, 633 N St Clair St Suite 2000, Chicago, IL 60611 USA; 3 Institute for Sexual and Gender Minority Health and Wellbeing, Northwestern University Feinberg School of Medicine, 625 N Michigan Ave Suite 1400, Chicago, IL 60611 USA; 4 Syndemic Infectious Disease Bureau, Chicago Department of Public Health, 333 South State Street Room 200, Chicago, IL 60604 USA

**Keywords:** Innovation tournament, Implementation mapping, HIV, PrEP, Participatory design methods, Sexual minority men

## Abstract

**Background:**

Participatory design approaches can improve successful selection and tailoring of implementation strategies by centering the voices of key constituents. To reduce incidence of the human immunodeficiency virus (HIV) in the USA, co-design of implementation strategies is needed for long-acting injectable cabotegravir (CAB-LA), a new form of HIV pre-exposure prophylaxis, among the disproportionately impacted population of sexual minority men (SMM). This manuscript describes the protocol for participatory design approaches (i.e., innovation tournament and implementation mapping) to inform implementation of CAB-LA among SMM (≥ 12 years), particularly Black and Latino populations, in Chicago.

**Methods:**

This research incorporates innovative methods to accomplish two objectives: (1) to crowdsource ideas for the design of implementation strategies for CAB-LA through a virtual innovation tournament and (2) to leverage the ideas from the innovation tournament to operationalize implementation strategies for CAB-LA thorough the systematic process of implementation mapping. A committee of constituents with diverse expertise and perspectives (e.g., SMM, implementation scientists, HIV clinicians, public health leadership, and community partners) will provide input throughout the design process.

**Discussion:**

This research will produce a menu of co-designed implementation strategies, which can guide plans for CAB-LA integration in Chicago and provide insights for other EHE regions. Further, as the first innovation tournament focused on HIV prevention, this research can provide a framework for participatory approaches across the care continuum. Given that the co-design of implementation strategies often does not involve the participation of individuals with lived experiences, this work will center the voices of those who will benefit most.

Contributions to the literature

- CAB-LA is a new form of long-acting, injectable pre-exposure prophylaxis for HIV prevention.
- This research will use innovative participatory methods to crowdsource ideas and operationalize strategies for the implementation of CAB-LA from target users and multidisciplinary constituents.
- Results will provide a menu of co-designed implementation strategies for CAB-LA integration to guide local implementation and provide insights for other regions.

## Background

Implementation science provides rigorous, systematic approaches to facilitating the integration of evidence-based interventions into practice [1]. Successful implementation requires targeted selection and tailoring of implementation strategies. Participatory design approaches offer collaborative methods for this process. Specifically, participatory design approaches directly involve end-users into the design of new products, for example [2]. Participatory design approaches facilitate the inclusion of the critical input of key constituents into the co-creation of implementation strategies. Thus far, the majority of studies have focused on the implementer’s perspective and behavior change, and there is an opportunity to better incorporate the perspectives and center the voices of the intended recipients of evidence-based practices.

Innovation tournaments offer a promising participatory design approach to incorporate end-users’ perspectives. Innovation tournaments generate new solutions to complex problems by crowdsourcing ideas for implementation [3–6]. They follow a “bottom-up” approach that allows end-users to provide ideas, followed by a structured process of evaluation. Literature has demonstrated the effectiveness of this participatory approach in generating novel solutions to intractable problems in healthcare [5, 7]. Further, innovation tournaments can produce input for the design of implementation strategies [5, 8]. A commonly used approach in implementation science, implementation mapping facilitates the operationalization of implementation strategies through a systematic approach incorporating constituent input, theory, and context [9]. Typically, implementation mapping begins with a needs assessment that involves contextual inquiry. There is a prime opportunity to use innovation tournaments to generate ideas from target users as input into the implementation mapping process.

### Research-to-practice gap in HIV prevention

Incident HIV cases in the USA continue to occur [10]. In response, the Department of Health and Human Services created the Ending the HIV Epidemic (EHE) Plan to focus on prioritized areas to reduce the number of new HIV infections by 90% [11]. Chicago, Cook County is one of the EHE prioritized jurisdictions [12]. In Chicago, HIV disproportionately impacts individuals from minoritized populations, including sexual minority men (SMM), Black individuals, and Latino individuals [13]. Reducing HIV incidence in Chicago requires concentrated efforts to implement evidence-based practices for HIV prevention, particularly among these key populations.

However, people with and vulnerable to HIV experience overlapping epidemics, or syndemics, that create barriers to the provision of HIV prevention options [14, 15]. Syndemic theory refers to the co-occurrence of multiple health conditions, which, in turn, increases disease burden [16]. Individuals with HIV experience multiple comorbid health problems, including psychiatric disorders, substance use disorders, sexually transmitted infections, and non-communicable diseases [17, 18]. This relationship is bidirectional, as syndemics also increase the risk of HIV acquisition [19]. Syndemics exacerbate health inequities and create competing demands for care. For example, psychiatric disorders create unique barriers for accessing HIV care (e.g., stigma) [18]. Therefore, successful implementation of HIV prevention requires attention to the unique determinants and syndemic issues faced by individuals vulnerable to HIV, such as the integration of services into existing care settings.

Long-acting injectable cabotegravir (CAB-LA) offers a novel form of HIV prevention. CAB-LA is an evidence-based version of pre-exposure prophylaxis (PrEP) [20, 21]. CAB-LA involves two initial intramuscular injections administered 4 weeks apart, followed by a dose every 8 weeks. Studies demonstrated high effectiveness, yielding a 79% relative reduction in HIV risk compared to daily oral PrEP [22]. In 2021, the United States Food and Drug Administration (FDA) approved the use of CAB-LA for adolescents (age 12 years and older) and adults [23]. Given the barriers to daily oral PrEP uptake and adherence in Chicago (e.g., daily-dose regimen, stigma, and competing needs) [24–26], CAB-LA offers a potentially more acceptable and feasible option for HIV prevention. To maximize the likelihood of successful integration of CAB-LA in Chicago, co-design of implementation strategies is critical.

This manuscript outlines the protocol to co-design strategies for the delivery of CAB-LA among adolescent and adult SMM in Chicago, with particular attention to Black and Latino populations. To accomplish this goal, this research will pursue the following objectives: (1) to crowdsource ideas for the design of implementation strategies for CAB-LA through a virtual innovation tournament and (2) to leverage ideas from the innovation tournament to operationalize implementation strategies for CAB-LA thorough the systematic process of implementation mapping.

## Methods

**Fig. 1:**
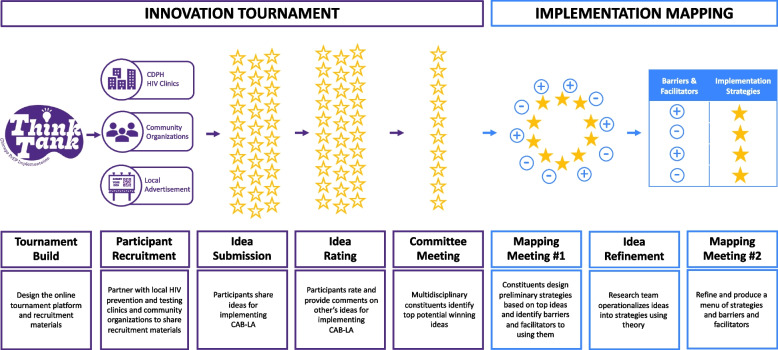
Overview of the research study

Figure 1 provides an overview of the research. Briefly, the innovation tournament will follow a three-step process: (1) participant submission of ideas, (2) participant voting on ideas, and (3) evaluation of ideas by a multidisciplinary group. The innovation tournament will produce top ideas that will serve as the input for the design of strategies in implementation mapping. The implementation mapping process will involve two meetings of key constituents to identify potential determinants of implementation and specific actions for a final menu of implementation strategies.

### Study team and governance

A public-academic partnership with transdisciplinary methodological expertise (e.g., implementation science, participatory methods, and community-engaged research) and content expertise (e.g., HIV, sexual and gender minority health, and public health) will lead this study. The team includes academic investigators and leadership from the Chicago Department of Public Health (CDPH). In addition, recruitment for study activities will involve collaboration with community-based organizations. The Institutional Review Board (IRB) at Northwestern University serves as the primary IRB, where approval was granted on August 1, 2023.

### Study design

**Fig. 2:**
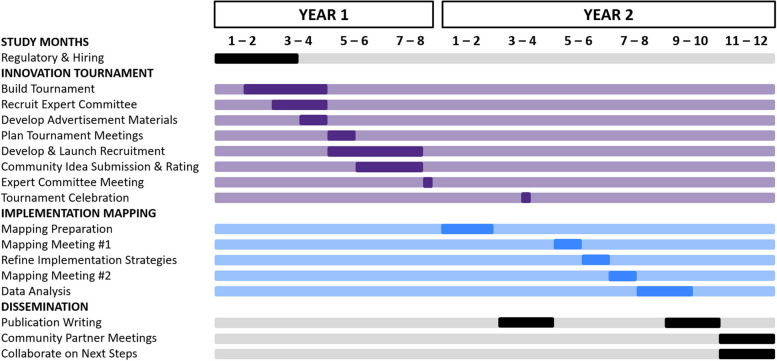
Research timeline

The study will employ the participatory approach of an innovation tournament and the systematic approach of implementation mapping. The research will occur over the course of 2 years (September 2023–April 2025). Figure 2 provides an outline of the full study timeline. Briefly, year 1 will focus on regulatory activities, team hiring, and the innovation tournament (e.g., build, data collection, and selection of top ideas). Year 2 will focus on the implementation mapping process (e.g., meetings, implementation strategy refinement, and data analysis) and dissemination of findings (e.g., development of publications, community partner meetings, and collaboration on next steps for the strategies).

### Aim 1 Innovation tournament

To systematically include the input and voice of key constituents in the design process, the research team will deploy an innovation tournament to crowdsource ideas for implementation strategies for CAB-LA.

#### Participants and recruitment

Participants for the innovation tournament will include target users (i.e., SMM) 13 years and older based on FDA approval for CAB-LA [23]. For this research, SMM refers to gay, bisexual, and other men who have sex with men, inclusive of all individuals with male gender identity regardless of sex assigned at birth. Recruitment will focus on SMM broadly, but efforts will concentrate on Black and Latino SMM due to the disproportionate impact of HIV and inequity in access to HIV prevention among these populations. Procedures will involve multiple steps. A Chicago-based graphic designer will collaborate with the research team to develop advertisement materials for dissemination in a variety of platforms. All deliverables will be available in English and Spanish. Partnership with community-based organizations serving the lesbian, gay, bisexual, transgender, and queer (LGBTQ), Black, and Latino communities will help facilitate promotion and ensure reach to the target population. In addition, collaboration with the CDPH network of 18 clinics that provide HIV prevention services to approximately 60,000 patients in the Chicago eligible metropolitan area will increase reach to individuals vulnerable to HIV. Advertisement will include social media posts, distribution of links via email, physical fliers in public spaces (e.g., LGBTQ arts center) and local businesses (e.g., LGBTQ bars and coffee shops), and public transportation advertisements. In addition, to reach individuals who may not have access to technology and the online innovation tournament platform, a member of the research team will visit community partner sites with a tablet for in-person recruitment. Recruitment strategies will cover a geographically diverse region of the city based on the location of the partner organizations and clinics. Further, review of the geospatial distribution of HIV cases and geospatial distribution of Black and Latino populations in Chicago will guide selection of flier posting and transportation campaigns. To incentivize participation, 15 randomly selected participants will receive a $250 gift card.

#### Innovation tournament

The research team will partner with website developers to build a custom platform to host the virtual innovation tournament. The platform will be available in English and Spanish. The homepage of the tournament will describe the purpose of the study and an option to participate. After providing informed consent and demographic information, participants will respond to a prompt that asks for their ideas on how to get CAB-LA to SMM (12 years and older) in Chicago. The prompt will probe for ideas on implementation in diverse care settings (e.g., places where they receive HIV prevention/care, primary care, or mental health care). The platform will allow for the submission of a detailed description of the idea. All ideas will be submitted anonymously. Participants will be allowed to submit multiple ideas. Upon submission, participants will have the option of viewing other previously submitted ideas and providing feedback via comments and a 5-point scale rating. Comments and ratings from other participants will not be visible to participants at the time of providing feedback. To prevent inappropriate comments, the research team will review the submissions each day before approving the publication of the live post. The tournament will stay open for 2 months to collect as many ideas as possible. The research team will monitor the number of submissions during weekly investigator meetings and adjust recruitment strategies as needed to ensure successful recruitment, with regard to the number of submissions and participant demographics (i.e., Black and Latino SMM).

#### Selection of top ideas

The research team will form a “challenge committee” to select the top ideas. The committee will comprise seven multidisciplinary constituents (e.g., implementation scientists, HIV clinicians, CDPH leadership, and community partners). Recruitment will target individuals in Chicago to increase familiarity with the context and individuals from diverse settings (e.g., community-based organization, academic institution, and local health department). The challenge committee will meet to evaluate submitted ideas based on novelty, feasibility, and potential impact on facilitating implementation of CAB-LA among SMM. All ideas will be presented to the challenge committee in advance of the discussion. This process will result in a ranking of ideas. Participants with the top ideas will receive a $250 gift card and participate in an in-person celebration. The challenge committee will receive an honorarium for their time.

#### Outcomes

The innovation tournament will generate potential solutions (i.e., winning ideas), which will serve as one source of input into the implementation mapping process. Further, the participatory approach will foster community engagement.

### Aim 2 Implementation mapping

To systematically include partner input and theory into the design process, the research team will conduct implementation mapping to operationalize implementation strategies for CAB-LA.

#### Participants

Participants will include a multidisciplinary group of constituents (*N* = 14–16), including implementation scientists, representatives from community-based organizations, CDPH leadership, syndemics experts, HIV prevention experts, clinicians, and SMM. Members of the challenge committee that selected top ideas in the innovation tournament will be invited to participate and represent the respective areas of expertise. In addition, depending on the nature of the top ideas from the innovation tournament, additional perspectives may be recruited (e.g., pharmacists if ideas emphasize implementation in pharmacies). Recruitment will focus on the greater Chicago area to increase familiarity with the implementation context and the feasibility of in-person participation. Potential participants will be contacted via email to explain the purpose of the study. SMM participants ≥ 18 years with the top two ideas from Aim 1 will be invited to participate as well. Participants will receive an honorarium for their time.

#### Procedures

The implementation mapping process will involve multiple steps [9, 27]. First, the research team will develop a preliminary set of implementation strategies leveraging the top ideas from the innovation tournament and scientific literature (e.g., the Expert Recommendations for Implementing Change taxonomy [28], a set of 73 discrete implementation strategies). Second, the research team will convene a half-day, in-person meeting with all participants. The meeting will begin with brief presentations that provide an overview of the project, details of CAB-LA, and goals of the implementation mapping process. In addition, the presentation will include an explanation of syndemic theory [16] to support the group in identifying implementation determinants and designing strategies that address syndemic issues people vulnerable to HIV experience. Third, participants will divide into four small groups, during which a member of the research team will share the preliminary set of implementation strategies. Participants will have the opportunity to provide feedback on needed adaptations to the proposed strategies. In addition, participants will share perspectives on barriers and facilitators to implementation (e.g., individual factors such as attitudes or inner setting factors such as availability of resources) [29]. Fourth, at the conclusion of the first mapping meeting, the research team will synthesize the notes from the small group discussions to refine the implementation strategies into concrete actions. This step will translate the input into an implementation research logic model to link the identified implementation determinants and implementation strategies through theories of behavior change. Fifth, the research team will convene an additional half-day, in-person meeting with all participants. The meeting will begin with a presentation of the operationalized list of implementation strategies and overview of the goals for the session. Similar to the first meeting, participants will have the opportunity to provide feedback on the refined strategies in small group discussions. Sixth, the research team will synthesize participants’ feedback and incorporate any newly proposed implementation strategies. If differences for adolescents and adults arise, recommendations for implementation strategies will be separated into two lists.

#### Outcomes

The implementation mapping process will produce a menu of partner-informed strategies for implementing CAB-LA in Chicago, as well as potential barriers and facilitators to implementation. In addition, the elicitation of determinants combined with the strategies will provide input for the design of an implementation research logic model [30] to guide future research.

### Dissemination plan

Dissemination will comprise deliverables to reach constituents at multiple levels. First, dissemination will involve traditional scholarly output (e.g., peer-reviewed manuscripts and presentations at academic conferences). Given potential insights for both the HIV and implementation research communities, deliverables will target a diverse set of platforms and audiences. Second, to increase access to the research, dissemination will include the development of lay publications (e.g., blog posts and op-eds) and facilitation of presentations for local HIV partners. In addition, the research team will query partners for additional pathways to communicate information to the target users (e.g., presentations at local events), a key component for community-engaged research. Fourth, to facilitate communication to policymakers, dissemination will involve the development of a policy brief for local public health leadership. This brief will summarize the research and recommend next steps toward implementing and evaluating strategies.

## Discussion

This study includes multiple innovations. First, this research will leverage participatory approaches and methods from implementation science, which have only recently guided the integration of evidence-based practices in the HIV care continuum [31]. Based on a review of the literature, this is the first study to utilize an innovation tournament to elicit end-user (i.e., SMM) input to design strategies for the implementation of CAB-LA. In addition, the use of implementation mapping to develop a menu of implementation strategies will offer innovative methods that incorporate partner input, theory, and context. Given that design of most strategies to reduce new HIV cases do not involve the active participation of individuals vulnerable to HIV, the planned methods will center the voices of those who will benefit most. Second, syndemic theory guides the research, so this work will develop implementation strategies that respond to the unique determinants and syndemic issues SMM vulnerable to HIV experience. Third, this work will elicit ideas from and develop strategies for both adult and adolescent SMM, which will increase the reach of future implementation efforts.

This research has the potential to yield multiple public health benefits. The innovation tournament and implementation mapping processes will produce a set of multi-level, integrated strategies to implement CAB-LA for SMM. Results will guide plans for implementation of CAB-LA in Chicago, as well as provide insights for other EHE regions. In addition, this is the first innovation tournament focused on HIV prevention. This study can provide a framework for participatory approaches for other interventions in the HIV care continuum. Further, this research will complete the first steps in the pipeline to Ending the HIV Epidemic through the preparation for successful implementation of CAB-LA. By providing evidence-based prevention, key populations vulnerable to HIV will experience a reduced risk of infection (Chicago EHE pillar 3) [32]. This effort will reduce the risk of HIV transmission among the population, which, in turn, will reduce HIV incidence and contribute to Ending the HIV Epidemic in Chicago.

## Data Availability

N/a.

## Abbreviations

CDPH: Chicago Department of Public Health
EHE: Ending the HIV Epidemic
HIV: Human immunodeficiency virus
CAB-LA: Long-acting cabotegravir
PrEP: Pre-exposure prophylaxis
SMM: Sexual minority men
